# Impact of Self-Compacting Concrete Admixtures on Frost Resistance and Compressive Strength—Commensurability of Frost Resistance Criteria

**DOI:** 10.3390/ma14112922

**Published:** 2021-05-28

**Authors:** Adam Piekarczyk, Beata Łaźniewska-Piekarczyk

**Affiliations:** 1 Department of Building Structures, Faculty of Civil Engineering, Silesian University of Technology, 44-100 Gliwice, Poland; 2 Department of Building Processes and Building Physics, Faculty of Civil Engineering, Silesian University of Technology, 44-100 Gliwice, Poland

**Keywords:** concrete admixtures, non-destructive testing, internal frost resistance methods, frost resistance criteria, compressive strength

## Abstract

The article presents the results of original and relevant tests from the point of view of using self-compacting concrete admixtures, especially their compatibility with the cement and mutual compatibility in the case of using several admixtures in one mixture. The research contributes to the recognition of the effect of an unintentionally air-entraining superplasticiser (SP), anti-foam (AFA), viscosity-modifying (VMA) and air-entraining (AEA) admixtures on the internal frost resistance and compressive strength of self-compacting concrete. Positive and undesirable effects of the combined use of several admixtures in this area have not been the subject of extensive analyses and publications so far. Superplasticiser, which unintentionally introduced a large amount of air to the concrete mixture, had a negative effect on the strength of the concrete and a positive effect on frost resistance. The addition of AFA to such concrete did not change the strength but worsened the values of the parameters estimating frost resistance. The AEA admixture resulted in a decrease in the strength of concrete but contributed to a change in the tendency to weaken the frost resistance observed in non-air-entrained concrete. The article also deals with the problem of compliance of the frost resistance criteria estimated upon various measures. It may be disturbing that finding frost resistance based on one criterion does not always mean frost resistance on another criterion. The discrepancies can be significant and misleading.

## 1. Introduction

Frost-resistant concrete, including self-compacting concrete (SCC), should have an appropriate porosity structure. Intentional aeration is achieved by the use of air-entraining admixtures. Air-entraining agents have a foaming effect and, most of all, they stabilise the introduced air, providing the appropriate structure (distribution and size) to the air microbubbles. The bubbles act as stress-compensating chambers. Water freezing in the capillaries can press into the empty bubbles, which prevents the concrete structure from bursting. The microbubbles interrupt the continuity of the capillaries in the concrete, which prevents capillary and osmotic transport of water. As a result, the mechanisms mentioned above make the concrete resistant to the damaging effects of frost. Unfortunately, other admixtures besides air entrainment, the necessity to obtain the proper consistency of SCC concrete or anti-segregation such as viscosity-modifying admixtures (VMA) [1,2], are not indifferent due to the resulting air content. Currently, there are many manufactured admixtures and large variability of their properties within one type understood as a group of chemical products whose impact on a given property or several features of a concrete mix and/or hardened concrete is known. However, it is not possible to predict all the effects of admixtures or the undesirable effects. Among others, publications [3,4,5,6,7] analysed the influence of admixtures, mainly air-entraining ones, on the frost resistance of concrete.

A side effect of using some superplasticisers may be the introduction of a significant amount of air into the concrete mix. Industrially produced superplasticisers of this type often contain a significant amount of unreacted macromonomer (about 10%). Therefore, a substantial foam generation mechanism is common. Excessive air entrainment is mainly caused by the reduction in the surface tension of the liquid phase in the cement paste. Standard requirements [8,9] for chemical admixtures for concrete limit the increase in air content in the mix because of the addition of superplasticiser to 2% compared to the mix without admixture. The test results from Ref. [10] confirm that the new generations of superplasticisers have a negative air-entraining effect. The pores in the hardened concrete reach a diameter of more than 1 mm. Such unintentional air entrainment of concrete can be associated with both positive and negative consequences. The quantitative impact of this type of superplasticisers (SP) on the analysed parameters of hardened concrete is not broadly described in the literature. There is an analysis of the impact of “air-entraining” SP on concrete features other than those discussed in this paper [11,12,13,14].

A self-compacting concrete mix should have good flowability and, at the same time, a viscosity that will resist aggregate segregation during and after the mix is placed. It is possible to use an additive in the form of a modifier regulating the internal consistency of the concrete mix, commonly known as viscosity-modifying admixture (VMA), which increases the viscosity of the cement paste without the need to reduce the water content or increase the content of the dust fraction [15,16]. VMA are usually water-soluble polysaccharides, forming a network of long-chain particles, maintaining the homogeneity of the concrete mix [1,2]. The high potency of these admixtures makes it challenging to use them, related to dosing and side effects, such as the possibility of an increase in air content in the mixture due to less effective deaeration. According to the article [17], viscosity-modifying admixtures (VMA) may increase the demand for air-entraining admixtures, allowing the optimal amount of air in the concrete mix to be achieved. The increased VMA content increases the amount of water that can be associated with the polymer. As a result, less free water is available to the AEA, and as the amount of VMA increases, more AEA is needed.

The test results [18] confirmed the increase in air content along with the increase in the amount of VMA. The increase in the amount of air caused by VMA was 1.4, 2.5, 3.5 and 4.0%, respectively, at 0.01, 0.0275, 0.045 and 0.08% of the applied admixture in relation to the amount of water in m^3^ of the mixture. In addition, VMA admixtures do not indifferently affect the strength of concrete, which is also related to its frost resistance.

The effect of the VMA addition on the compressive strength of concrete is described in [19]. In concretes with the addition of VMA, reductions in strength were reported after 28 days (up to 5%) compared to concretes without the viscosity-modifying substance.

In other tests [20], slightly higher compressive strengths of concretes containing VMA were obtained. Compared to concrete without the modifier, the strength of concrete with VMA was higher by 15%. The higher content of VMA meant that the compressive strength was also higher than in the case of concrete without VMA, but the increases were smaller—up to 8%.

An anti-foam agent (AFA) can be used to reduce excessive air content in self-compacting concrete. AFA causes destabilisation, breaks the surface of air bubbles into the foam and causes coalescence. AFA influences the structure and distribution of pores in the concrete [21,22]. However, when using AFA, there is a problem with the compatibility of the superplasticiser–anti-foam admixture system [23].

The experimental studies presented in this article aimed to determine the effect of the application of chemical admixtures with different effects in self-compacting concrete and their mutual interaction. The research concerned two types of superplasticisers (SP), including one that caused an unintentional air-entrainment of the concrete mix, anti-foaming (AFA), air-entraining (AEA) and viscosity-modifying (VMA) admixtures. The analysed properties of concrete were internal frost resistance and compressive strength. The information in [24] on the frost resistance of high-strength, self-compacting, non-air-entrained concrete was also verified. Additionally, the results of testing internal frost resistance using three methods prompted the authors to discuss the equivalence of the criteria for assessing this parameter. The authors intended to check whether it is possible to estimate the frost resistance of non-air-entrained and air-entrained concrete, modified with various additional admixtures, using the non-destructive method.

Moreover, a question was asked whether the influence of additional admixtures on the porosity characteristics of concrete would be consistent with the results of its frost resistance, determined by the destructive and non-destructive method? For this purpose, the porosity characteristics of self-compacting non-air-entrained and air-entrained concrete, modified with additional AFA and VMA admixtures, were determined and then compared with the frost resistance of concrete estimated by the methods mentioned above. Finally, it was checked whether the non-destructive method allows for quantitative estimation of the frost resistance of self-compacting concrete modified with several simultaneously used admixtures.

## 2. Materials and Tests Methodology

### 2.1. Tested Concretes

Seven types of self-compacting concrete were tested. Their basic composition is summarised in Table 1. Individual concrete differed in the types of admixtures used to make the mixtures. The concrete symbols used in the further description of the test results, the types of admixtures and the explanation of their effect are given in Table 2. Table 3 contains information on the dosage of the admixtures used. The consistency of concrete mixes and air content, determined following the requirements of the relevant standards, is presented in Table 4.

### 2.2. Measures and Criteria of Concrete Internal Frost Resistance

Several different measures and criteria are used to assess the internal frost resistance of concrete. Ultrasonic non-destructive methods are very popular. Based on the measurement of the parameters of the ultrasonic pulse induced on the surface of the specimen at one point and received at another, the dynamic modulus of elasticity of concrete *E*_d_, can be estimated. The dynamic modulus of elasticity is a mechanical feature of concrete that is very sensitive to changes caused by the formation of macro- and microcracks [28]. In frost resistance testing, the damage is related to stress caused by hydraulic or osmotic pressure, which locally exceeds the tensile strength of concrete [29]. The value of *E*_d_ is expressed by

(1) $$E_{d}=\frac{1}{t^{2}}l^{2}\rho A_{\nu }$$

where *t* is the propagation time of the ultrasonic wave over a length of *l*, *ρ* is the density of the medium, while *A_v_* is a factor depending on the Poisson’s ratio.

The values of the dynamic modulus of elasticity can be determined in two ways. The first one is related to measuring the ultrasonic pulse transit time between the point at which the ultrasonic wave was induced and received (*E*_d.UPTT_). The second method is based on the measurement of fundamental resonant frequency (*E*_d.FF_) of concrete specimens vibrating using a modal hammer and recorded by an accelerometer. *E*_d.FF_ tests can be performed according to the ASTM C 215 [30] procedures. Changes in the dynamic modulus of elasticity *E*_d.FF_, along with the number of freezing and thawing cycles of concrete, can be used directly to assess frost resistance, which was performed in the studies described, for example, in [31,32,33].

More often, the relative dynamic modulus of elasticity *RDM* is used as a measure of concrete frost resistance, which is defined as the ratio of the dynamic modulus value determined after *N* freezing and thawing cycles *E*_d.N_ to the initial value determined before the frost resistance test *E*_d.0_, i.e.,

(2) $$RDM=\frac{E_{d.N}}{E_{d.0}}$$

After taking into account Formula (1), the value of the relative dynamic modulus of elasticity calculated based on the change of the transition time of the ultrasonic pulse *RDM*_UPTT_ can be determined in per cent from the formula

(3) $$RDM_{\mathrm{UPTT}}={\left(\frac{t_{0}}{t_{N}}\right)}^{2}\cdot 100\%,$$

where *t*_0_ is the time of transition of the ultrasonic pulse before freezing of concrete specimens, while *t_N_* is the time of wave transition after *N* freezing and thawing cycles between the same points on the specimen surface.

If the measured parameter is the fundamental resonant frequency, the relative dynamic modulus of elasticity, *RDM*_FF_, is determined. The values of the relative dynamic modulus of elasticity, *RDM*_UPTT_, are used to estimate concrete frost resistance in the CEN/TR 15177 [34] standard and were used in the tests described in [35,36]. *RDM*_FF_ is used to determine frost resistance following CEN/TR 15177 [34], ASTM C 666 [37] standards and was used, among others, in the studies presented in [4,33,36,38,39].

Among other parameters used to assess the frost resistance of concrete based on ultrasonic tests, one can also mention the so-called level of damage, *D*_FT_ [40,41], and a measure in the form of a relative change in the transition time of the ultrasonic impulse occurring in the standard RILEM TC-176 [42]. More advanced models based on the dynamic modulus of elasticity of concrete are also adapted [40,43,44].

In the research described in this article, two more methods of frost resistance assessment were used, which are included in the Polish annexe to the EN 206 standard [45]. The first one is the mean decrease in compressive strength of concrete specimens Δ*f*_F_ calculated as a percentage from the formula

(4) $$\Delta f_{F}=\frac{f_{F1}-f_{F2}}{f_{F1}}\;\cdot 100\%,$$

where *f*_F1_ is the mean compressive strength of reference specimens, not frozen, and saturated with water, while *f*_F2_ is the mean compressive strength of specimens, after the last thawing, saturated with water.

The second method is the mean weight loss after the test Δ*m*_F_, which is calculated as a percentage from the equation

(5) $$\Delta m_{F}=\frac{m_{F1}-m_{F2}}{m_{F1}}\;\cdot 100\%,$$

*m*_F1_ is the mean mass of the specimen before their first freezing, in a state of water saturation, while *m*_F2_ is the mean mass of the specimen after the freezing and thawing cycles, saturated with water.

The measures of concrete frost resistance must be associated with the appropriate criteria, i.e., limit values, after exceeding which it is assumed that the tested concrete is not frost-resistant.

When the relative dynamic modulus of elasticity *RDM*_UPTT_ (3) or *RDM*_FF_ is used as a measure of frost resistance, according to the criteria contained in the RILEM TC-176 [42], it is assumed that the concrete for which RDM <80% is not frost-resistant.

According to the Polish national annexe to EN 206 [45], it is assumed that concrete is not frost-resistant when the mean relative strength decreases, Δ*f*_F_ > 20% (4). If the mean weight loss of the concrete specimens, Δ*m*_F_ (5), in the form of damaged corners and edges, aggregate spalling, etc., exceeds 5%, the concrete is considered not to meet the frost resistance criterion.

There are also original criteria combining different properties of concrete. For example, in the article [46], a criterion combining the number of freezing and thawing cycles, *N*, with the value of the ratio of ultimate compressive stress to uniaxial strength, *σ*_3_/*f*, and the ratio of lateral stress restraining deformations of concrete to uniaxial strength, *σ*_2_/*f,* was proposed. In the study [47], the frost resistance has been associated with the concrete compressive strength, the volume of air from 0.8 to 6.5% and the concrete mix consistency.

### 2.3. Methodology

The tests were carried out on cuboidal specimens of hardened concrete with a length of 400 mm and a 100 × 100 mm cross-section and cubic specimens with a side length of 150 mm. Specimens of the first type (3 for each type of concrete—21 in total) were subjected to frost resistance tests and were used to measure the velocity of the ultrasonic pulse before freezing and after a designated number of freezing and thawing cycles, *N*. Cubic specimens (6 for each type of concrete—42 in total) were not subject to frost resistance tests. They were used for compressive strength evaluation after 28 and 128 days of curing in the state of saturation with water. The remaining cubic specimens (3 for each type of concrete—21 in total) were used to determine the compressive strength of concrete after 300 freezing and thawing cycles.

Ultrasonic wave transition time was measured in the tests conducted by the so-called direct transmission method. A 54 kHz pulse was generated and received using flat ultrasonic transducers applied to specimens on the opposite, frontal, and parallel surface sizes 100 × 100 mm. The ultrasonic transducers were coupled acoustically to the concrete surface using technical vaseline. The tests were carried out using a Proceq Pundit Lab + device with a resolution of the ultrasonic wave transition time of 0.1 μs until the lowest value of the pulse transit time was obtained, which indicated that the thickness of the coupling agent layer was reduced to the minimum.

All concrete specimens were demoulded after three days of curing at 20 °C. After removing moulds, cubic specimens not submitted to frost resistance tests were stored until testing in the water at 20 °C. After 28 and 128 days of curing, such specimens were removed from the water, wiped and compressed in a testing machine.

The pore distribution in hardened concrete after 28 days of curing was determined using the method specified in EN 480-11 [48]. Based on the measurements, the standardized parameters for the characterization of the microstructure of air voids was determined: spacing coefficient *L*, specific pore surface *α*, total air content *A*, the content of air voids with a diameter smaller than 300 μm *A300*.

The freezing and thawing procedure of the specimens, and the method of their preparation and testing, were following the requirements of the Polish standard PN-B-06265 [45], which is a national annexe to the EN 206 standard. Cuboid and cubic specimens, provided for cyclic freezing and thawing, were stored after being demoulded for 18 days at 18 °C in the air with relative humidity above 90%. After 21 days, the specimens were saturated with water. The specimens were placed in a container on 10 mm and 60 mm thick supports. Their bases do not touch the bottom of the vessel. Water at 18 °C was poured into the container to a level about 10 mm above the upper edge. This level was maintained until the end of concrete saturation. All specimens were removed from water every 24 h, wiped and weighed to the nearest 0.2% of their mass. The saturation of concrete with water was assumed to be when the masses of the specimens in two subsequent weighings did not differ by more than 0.2%. After 28 days of curing, the specimens saturated with water and weighed were placed in a freezing chamber. The temperature in the chamber was −18 °C at the time of placing the specimens. The distance between the specimens and between the specimens and the chamber walls were not less than 20 mm. Each freezing cycle at −18 °C lasted 4 h, followed by a four-hour thawing cycle of specimens in the water at 18 °C. Four hundred fifty such cycles were planned. Cuboid specimens after 25, 75, 100, 150, 250, 300, 325, 350, 400 and 450 cycles, after thawing were removed into the water, wiped and measurements of ultrasonic pulse transition time were carried out. Frozen cubic specimens after 300 test cycles were thawed, wiped, and weighed to the nearest 0.2% of their mass and compressed in a testing machine.

## 3. Tests Results

**Figure 1:**
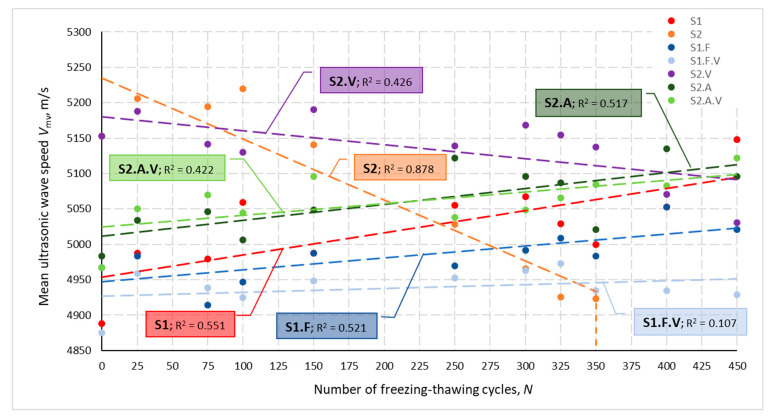
Measured ultrasonic wave velocity for individual concrete types depending on the number of freezing and thawing cycles.

Figure 1 shows the mean values of the ultrasonic pulse velocity *V*_mv_ for individual concrete types obtained after serial numbers of freezing and thawing cycles. Trends in velocity changes as the number of cycles increases were approximated by linear regression. Severe frost damage to the S2 concrete structure prevented ultrasonic testing after 350 freezing and thawing cycles. All recorded ultrasonic wave velocities obtained in the tested concretes ranged from 4689 to 5242 m/s, with a coefficient of variation for individual concrete types (in a group of three specimens) not exceeding 4.54%. However, a coefficient of variation greater than 1.50% was obtained only for S2 and S2.V concretes.

The highest initial mean ultrasonic velocity *V*_0.mv_ after 28 days of curing of 5153 m/s was obtained for non-air-entrained concrete S2 and S2.V. The lowest initial velocities in the range 4875 to 4967 m/s were recorded for concretes S1, S1.F and S1.F.V. For S2.A and S2.A.V concrete, initial velocities *V*_0.mv_ of 4983 and 4967, respectively, were obtained.

**Figure 2:**
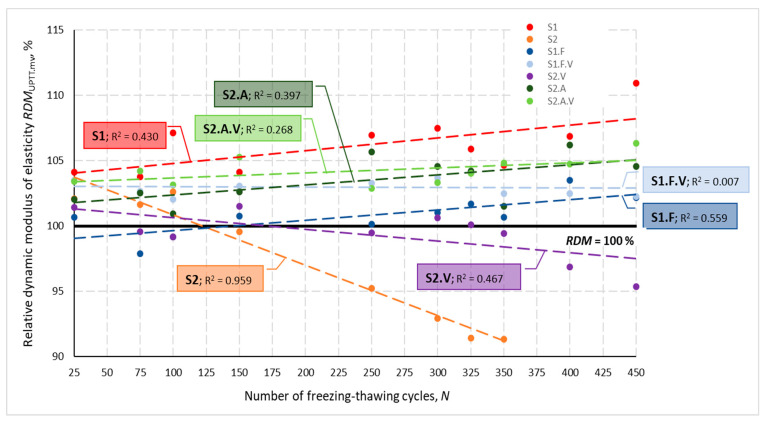
Mean relative dynamic modulus of elasticity for individual concretes after *N* cycles of freezing and thawing.

Figure 2 presents changes in the relative dynamic modulus of elasticity *RDM*_UPTT_ for all tested concretes depending on a number of freezing and thawing cycles *N*. Calculated based on the change in ultrasonic wave propagation time, the *RDM*_UPTT_ values ranged from 83.6 to 112.0%. The coefficient of variation *ν*_RDM_ determined for three specimens of each concrete after N cycles did not exceed 7.92%, with values higher than 4.0% obtained only for S2 and S2.V concrete. The mean values of the relative dynamic modulus of elasticity were higher than 100% except, again, specimens of S2 and S2.V and S1.F concrete until the number of *N* cycles reached 100.

The graphs shown in Figure 1 and Figure 2 also show that the lowest impact of freezing and thawing cycles *N* on the velocity *V*_mv_ and the *RDM*_UPTT._mv modulus was obtained for concretes containing VMA admixture. This is evidenced by the lowest values of the coefficient of determination R*^2^*.

Table 5 summarises the relative mass loss of concrete specimens Δ*m*_rel_ determined after *N* = 300 freezing and thawing cycles. The highest values were obtained for concrete S1.F and S1.F.V. In no case, the relative weight loss exceeded 1.0%. For other concrete types, it can be considered that no weight loss occurred or was negligible.

The mean compressive strengths of concrete determined on cubic specimens with a side length equal to 150 mm are shown in Table 6. The strengths of individual types of concrete obtained after 28 days, *f*_c.mv.28d_, and 128 days, *f*_c.mv.128d_, on specimens not subjected to frost resistance tests are summarised. The strength of reference cubic specimens frozen and thawed over 300 cycles, *f*_c.mv.300FT_, in the same manner and under the same conditions as the cuboid specimens used to tests the ultrasonic pulse transition time, is also given. The highest strength after 28 and 128 days of curing was demonstrated by S2 and S2.V concrete types, in which air was not introduced into the concrete mix (neither intentionally nor unintentionally). The opposite situation occurred in the case of specimens subjected to freezing and thawing. These concrete types had the lowest compressive strength after 300 cycles of frost resistance testing.

**Figure 3:**
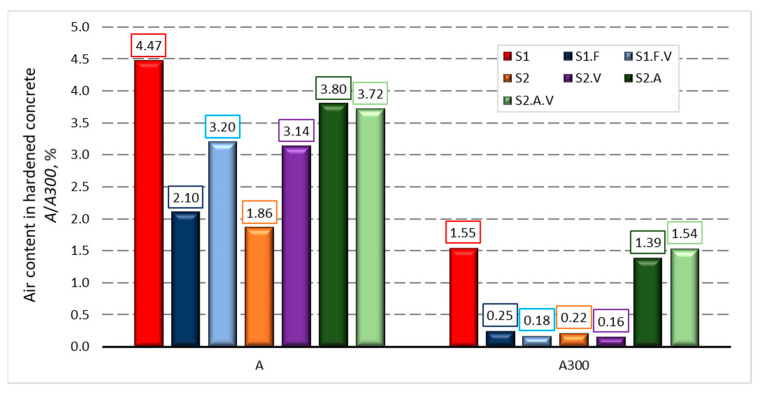
Results of measurements of the total volume of air voids, *A*, and the volume of voids, *A300*, with a diameter less than 300 μm in hardened concrete after 28 days of curing.

**Figure 4:**
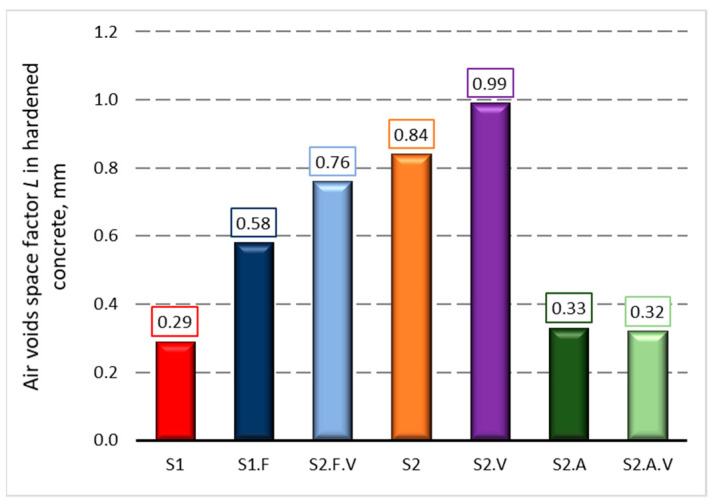
Air voids space factor *L* in hardened concrete after 28 days of curing.

**Figure 5:**
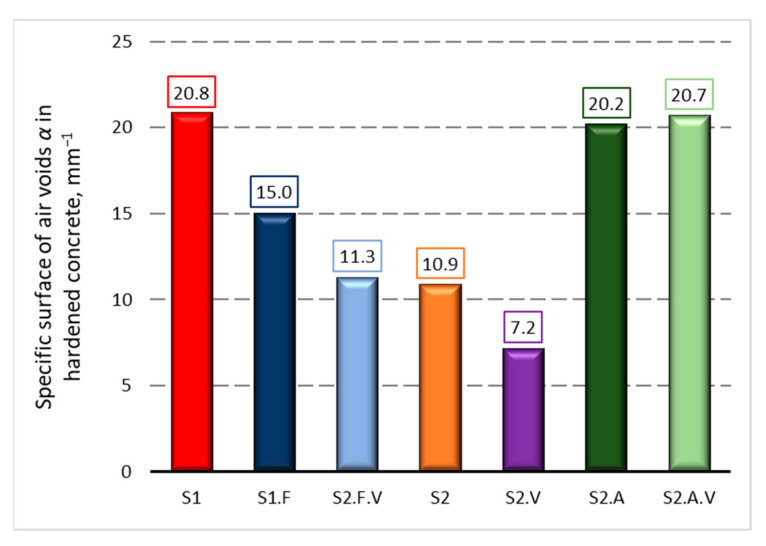
Estimation of specific surface of air voids *α* in hardened concrete after 28 days of curing.

Figure 3, Figure 4 and Figure 5 present the measurements of the concrete porosity parameters after 28 days of curing. The results indicate that the used admixtures significantly affect the porosity characteristics of self-compacting concrete, both air-entrained and non-air-entrained. Detailed analysis of the influence of admixtures on the microstructure of hardened concrete is presented in the publication [49].

## 4. Discussion of the Results

The tests have shown that the use of concrete admixtures (Table 2) with the same base composition (Table 1) affects the tested characteristics of concrete, which are the measures of their frost resistance.

**Figure 6:**
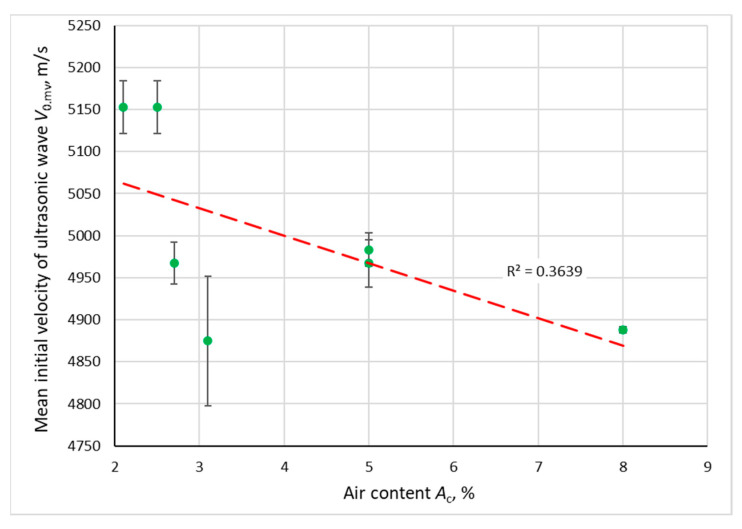
Dependence of the mean initial ultrasonic wave velocity (after 28 days of curing) on the air content in the concrete mix.

**Figure 7:**
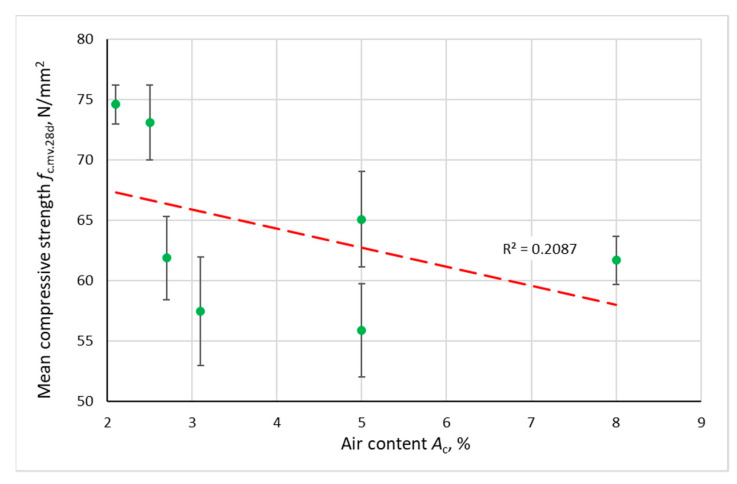
Dependence of the mean compressive strength of concrete after 28 days of curing on the air content in the concrete mix.

The relationship between the initial compressive strength of concrete, the velocity of the ultrasonic wave and the air content in the concrete mix is shown in Figure 6 and Figure 7. There is a trend towards a decrease in initial velocity *V*_0.mv_ and compressive strength *f*_c.mv.28d_ with an increase in the air content *A*_c_. Error bars represent a typical range of variation (*m*_X_ ± *s*_X_).

Table 7 summarises the increases in absolute, Δ*f*_c.abs.d_, and the relative, Δ*f*_c.rel.d_, mean compressive strength of concrete that was not subjected to freezing and thawing between 28 and 128 days of curing. This table also includes the absolute, Δ*f*_c.abs.300FT_, and the relative, Δ*f*_c.rel.300FT_, decreases in the mean compressive strength of reference specimens after 300 freezing and thawing cycles concerning the strength of non-frozen concrete after 128 days of curing.

**Figure 8:**
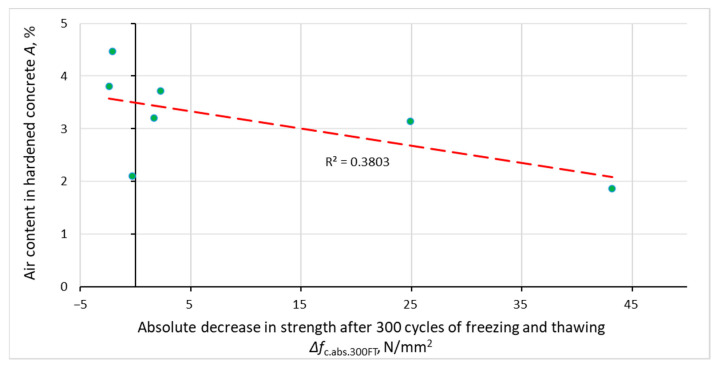
Dependence of the decrease in the compressive strength of concrete after 300 cycles of freezing and thawing on the total amount of air in the hardened concrete.

**Figure 9:**
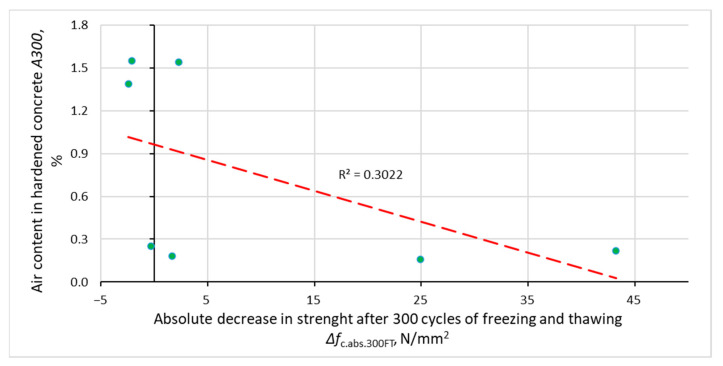
Decrease in the compressive strength of concrete after 300 cycles of freezing and thawing on the amount of air voids smaller than 300 μm in the hardened concrete.

**Figure 10:**
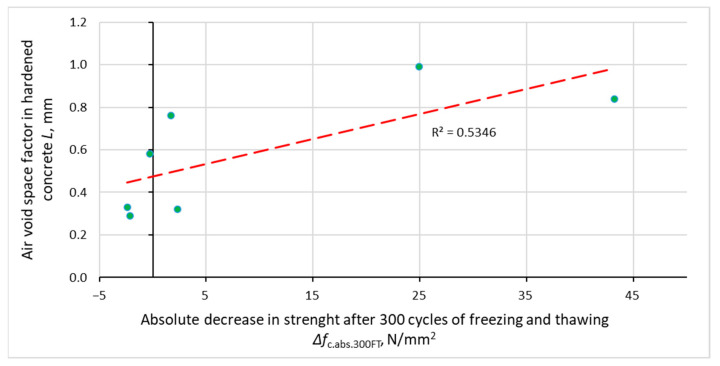
Relationship between air voids space factor in hardened concrete and reduction in concrete compressive strength after 300 cycles of freezing and thawing.

**Figure 11:**
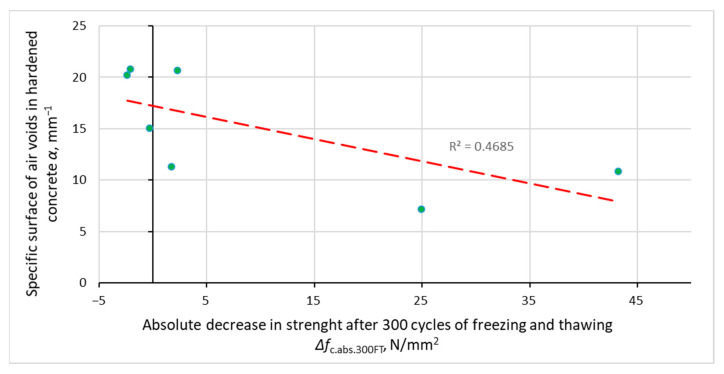
Dependence between specific surface air voids in hardened concrete and reduction in concrete compressive strength after 300 cycles of freezing and thawing.

Figure 8, Figure 9, Figure 10 and Figure 11 show the relationship between the parameters of concrete porosity and its strength reduction after 300 cycles of freezing and thawing. The analysis indicates the diversified effect of AFA and VMA admixtures on the reduction in its strength after the freeze–thaw cycles. With significantly different air content in intentionally and incidentally aired concrete, the compressive strength determined after the freeze–thaw cycles is comparable, except for non-air-entrained concrete (S2, S2.V), where a significant reduction in strength was noted.

Table 6 and Table 7 show the mean compressive strengths obtained for concrete specimens not subjected to frost resistance testing after 28 and 128 days of curing and the relative differences between these values Δ*f*_c.rel.d_. Increases in mean compressive strength were obtained in the range from 3.2 to 17.4 N/mm^2^ and relatively from 4.4 to 31.1%. The smallest increase in relative and absolute strength occurred in non-air-entrained concrete S2.V with viscosity-modifying admixture. The most significant growth in mean strength was obtained in the case of intentionally air-entrained concrete S2.A. In three types of concrete, S1, S1.F and S2.A, slight mean strength gains were obtained. For the following two concrete types, S1.F.V and S2.A.V, minor decreases were observed. The changes in the mean strength of S1.F.V and S2.A.V concrete did not exceed 2.4 N/mm^2^ and 3.3% and can be considered insignificant. The mean compressive strength of non-air-entrained S2 and S2.V concretes after 300 freezing and thawing cycles drastically decreased. The reductions in mean compressive strength for these concretes were 43.2 N/mm^2^ (49.3%) and 24.9 N/mm^2^ (32.6%), respectively.

Usually, the higher the ultrasonic wave velocity, the larger the compressive strength of the concrete [50,51,52]. It can be assumed that the velocity difference is associated with the difference in the compressive strength of concrete. The test results were used to check how the mean compressive strength of the analysed concrete is related to the changes in the propagation velocity of the ultrasonic pulse and the relative dynamic modulus of elasticity.

**Figure 12:**
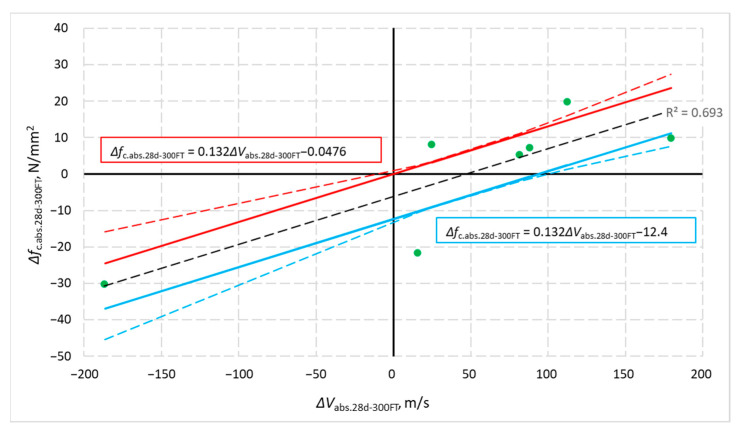
Correlation between absolute changes in mean compressive strength and ultrasonic pulse velocity obtained for concrete specimens after 300 cycles of frost resistance tests and not-frozen specimens of the same age.

**Figure 13:**
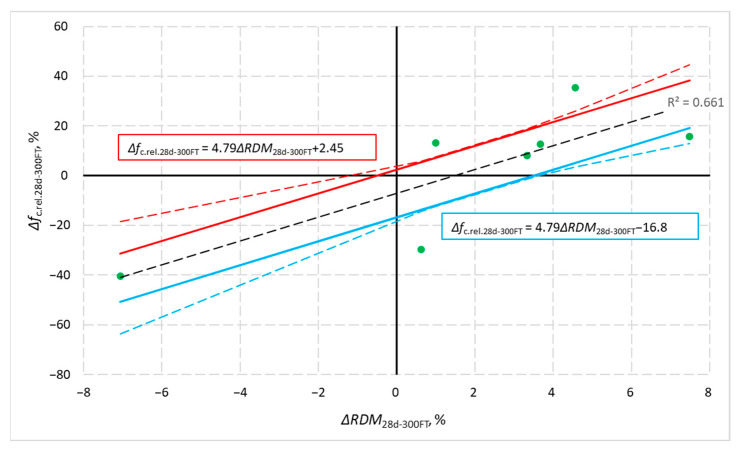
Relationship between the relative changes in mean compressive strength and the differences in the relative dynamic modulus of elasticity obtained from tests of concrete specimens after 300 cycles of freezing and thawing and not-frozen specimens.

Figure 12 shows the relationship between the mean absolute differences in compressive strength, Δ*f*_c.abs.28d-300FT_, and the mean changes in ultrasonic wave velocity, Δ*V*_abs.28d-300FT_, obtained after 28 days of curing of specimens not subjected to freezing and specimens after 300 cycles of frost resistance testing. Figure 13 illustrates the relationship between the relative changes in mean compressive strength, Δ*f*_c.rel.28d-300FT_, and the differences in the relative dynamic modulus of elasticity, Δ*RDM*_28d-300FT_, of concrete of the same age not subjected to freezing (in the case of Δ*RDM*_28d-300FT_ compared to the value on day 28, i.e., 100%) and after 300 cycles of freezing and thawing. The graphs (Figure 12 and Figure 13) contain test results as points with coordinates Δ*f −* Δ*V* and Δ*f −* Δ*RDM*. These graphs also include regression line, confidence interval curves (dashed red and blue curve) and regression lines shifted to these curves (thick red and blue lines). Confidence interval curves were determined to assume a continuous Student’s *t*-test probability distribution with n − 2 degrees of freedom and a 0.90 confidence factor. Therefore, the regression lines shifted to the confidence interval represents the relationship Δ*f −* Δ*V* and Δ*f −* Δ*RDM* with a 90% probability of occurrence.

### 4.1. The Comparison of Test Results of SCC Mix with the Addition of SP1 or SP2 Superplasticisers

The use of the SP1 superplasticiser based on polycarboxylates had the side effect of introducing a large amount of air into the mix (Table 4) and Figure 3.

The only additive used in the S2 mixture was the SP2 superplasticiser, which did not cause the undesirable effect of significant air entrainment. The measured air content in this mixture and hardened concrete was about 2%.

**Figure 14:**
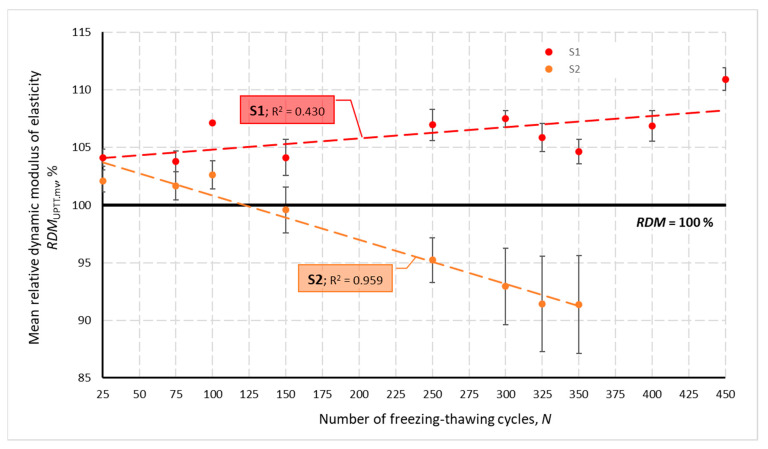
Mean relative dynamic modulus of elasticity for S1 and S2 concretes after *N* cycles of freezing and thawing.

According to two criteria for the assessment of frost resistance, i.e., the loss of mass of the specimens and the change in the relative dynamic modulus of elasticity *RDM*_UPPT_, concrete S2 could be considered frost-resistant:

- Value of *RDM*_UPPT_ after 300 cycles of freezing and thawing was 92.9% (however, after 350 cycles of frost resistance testing, the condition of the concrete was so bad that it was impossible to perform tests using the ultrasonic method)—Figure 2 and Figure 14;
- No loss in mass of the specimens was found;

Using the criterion of the change in compressive strength after 300 cycles, concrete S2 cannot be considered frost-resistant. The decrease in strength was as high as 49.3% (43.2 N/mm^2^)—Table 7.

Following the criteria for assessing the internal frost resistance, S1 concrete was considered frost-resistant with the methods used in this study, because

- There was no decrease in strength (3% increase was obtained)—Table 7;
- *RDM*_UPPT_ values were > 100% regardless of the number of freeze and thaw cycles—Figure 2 and Figure 14;
- No weight loss of the specimens occurred.

The compressive strength of the S2 concrete was the highest among all tested concretes. This applies to the strength determined after 28 and 128 days of curing (74.6 and 87.6 N/mm^2^, respectively)—Table 6 and Table 7. The increase in compressive strength between 28 and 128 days was 13.0 N/mm^2^ (17.4%). The highest achieved ultrasonic pulse velocity—5153 m/s (Figure 1) was also associated with the highest compressive strength after 28 days.

In the case of the unintentionally air-entrained concrete S1, in relation to concrete S2, the following conclusions can be drawn:

- Lower mean compressive strength was determined after 28 and 128 days of curing, by 13.0 N/mm^2^ (17%) and 18.2 N/mm^2^ (21%), respectively—Table 6;
- There was a lower strength gain between days 28 and 128—Table 7;
- There was a decrease in the initial velocity of the ultrasonic pulse by 5.1%—Figure 1.

A statistical test was used to conclude the influence of individual admixtures. Calculations associated with a Student’s *t*-Test determined whether two samples are likely to have come from the same two underlying populations that have the same mean. In the case of concretes S1 and S2, the statistical test results showed that the compressive strength after 28 and 128 days of curing and the strength and *RDM*_UPTT_ after 300 cycles of frost resistance test come from populations with different mean values. This proves that the influence of SP1 and SP2 superplasticizers on the parameters mentioned above is significant.

### 4.2. The Comparison of the Influence of AFA and VMA Admixtures on Air-Entrained SCC as a Side Effect of SP1

The concrete mix S1.F uses a superplasticiser SP1 that introduces a significant amount of air into the mix, and the anti-foam admixture AFA, whose task was to reduce air content. In this study, concerning concrete without AFA, the air content decreased by over 5% (Table 4) in the mix and near 2.5% in hardened concrete (Figure 3). The presence of fatty alcohol ester in the composition of AFA, which contributes to the high efficiency of anti-foams, also has a negative effect on the cement hydration process and may reduce the strength of hardened concrete. The tensile strength is also weakened, which was confirmed by the highest weight loss of specimens.

All three criteria for the assessment of frost resistance indicate that concrete S1.F was frost-resistant, because

**Figure 15:**
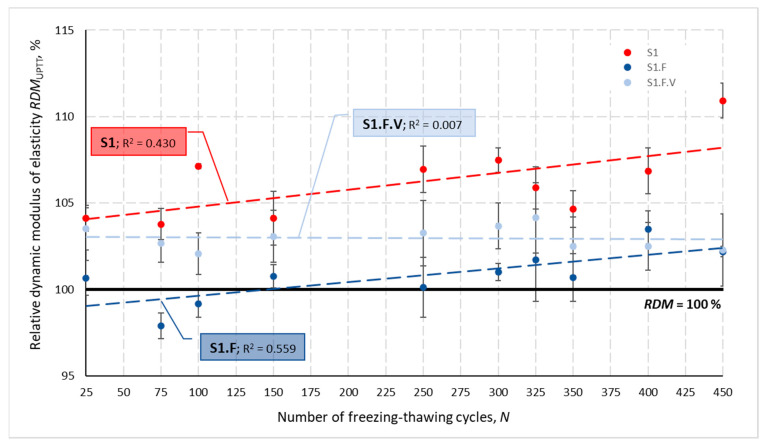
Mean relative dynamic modulus of elasticity for S1, S1.F and S1.F.V concretes after *N* cycles of freezing and thawing.

- There was no decrease in strength (a slight increase was obtained)—Table 7;
- *RDM*_UPPT_ values were > 100% when the number of cycles exceeded 150 cycles—Figure 2 and Figure 15;
- The weight loss of the specimens was less than 1%.

The reduction in the amount of air in the concrete resulted in the following, compared to S1 concrete without AFA:

- An increase in strength after the frost resistance test was smaller—Table 7;
- *RDM*_UPPT_ values were about 5% lower—Figure 15;
- Specimen weight loss increased from zero to less than 1%.

The use of AFA in concrete S1.F caused, compared to concrete S1, insignificant change to the 28- and 128-day compressive strength. Thus, the differences in the increase in strength between 28 and 128 days were also insignificant—Table 6 and Table 7. The initial velocity of the ultrasonic wave, determined after 28 days, was also slightly changed—Figure 1. This confirms the results of the strength tests. The compressive strength of concrete, in which the amount of air has been significantly reduced compared to S1 concrete without AFA, should visibly increase. However, this did not happen. This may confirm the negative influence of AFA on the cement hydration process.

The statistical test showed that the mean strengths after 28 and 128 days of curing and after 300 freeze–thaw cycles correspond to the population with the same mean values. This means that the influence of the AFA admixture added to S1 concrete is not significant from the point of view of the analyzed strength parameters.

Few studies have been published on the effect of AFA admixture on the internal frost resistance and strength of concrete. In studies [22], an increase in the compressive strength of concrete was obtained after 28 days of maturation after the application of AFA admixture. A slight increase in strength (9.3%) was observed in tests [21], with a decrease in air content in the concrete mix by about 60%. In the studies presented here, no increase in strength was obtained, despite a similar decrease in the amount of air. It is suspected that some AFA admixtures containing fatty alcohol ester may adversely affect cement hydration. The conclusions of the negative impact of AFA on the compressive strength of concrete are also presented in [23].

In the concrete mix S1.F.V, apart from the superplasticiser SP1 and AFA admixture, an admixture modifying the viscosity was introduced. SP1 and VMA additives can contribute to the high amount of air in the mixture. The results of testing the amount of air in the S1.F and S1.F.V mix (see Table 4, Figure 3) showed that the VMA used in the present tests did not significantly deteriorate the deaeration of the concrete mix. In the S1.F.V mixture, there was 0.4% more air than in S1.F and about 1% more in hardened concrete.

Based on the criteria used in the tests, the frost resistance of concrete S1.F.V was confirmed:

- There was a slight decrease in the strength by about 3%—Table 7;
- *RDM*_UPPT_ oscillated at about 103%—Figure 15;
- Weight loss slightly exceeded 0.5%.

The use of the VMA additive resulted in the fact that concerning concrete S1.F without this additive:

- There were slight drops in strength, instead of equally slight increases;
- *RDM*_UPPT_ values were higher—Figure 15.

The VMA admixture caused a slight decrease in strength determined after 28 and 128 days of maturation (up to 7% and 5%, respectively) and after 300 cycles of freezing and thawing (up to 9%) concerning concretes not containing VMA, i.e., S1 and S1.F. Strength losses may be due to slightly higher air content in hardened concrete containing VMA (Figure 3).

The Student’s *t*-test showed that the changes in compressive strength and RDM_UPTT_ after 300 cycles of frost resistance are accidental. This means that the VMA admixture did not significantly affect the strength and frost resistance of unintentionally air-entrained concrete.

Unfortunately, the authors were unable to find any publications on the study of the effect of VMA admixture on the mechanical parameters of concrete.

### 4.3. VMA and AEA Impact in Case of Non-Air-Entrained SCC

The intentional air-entraining of the concrete mix, which contained only a superplasticizer, caused the expected and commonly known effects. The positive effect was the improvement of the frost resistance of concrete, while the negative effect was the reduction in concrete compressive strength. This was confirmed by the results of tests of concrete S2.A and S2.A.V with the addition of AEA concerning the parameters of concrete S2 and S2.V without this admixture. As a result of adding AEA to mixtures S2 and S2.V, the air content increased from 2.1 and 2.5% to 5.0% (Table 4). This difference was smaller in the hardened concrete (Figure 3).

**Figure 16:**
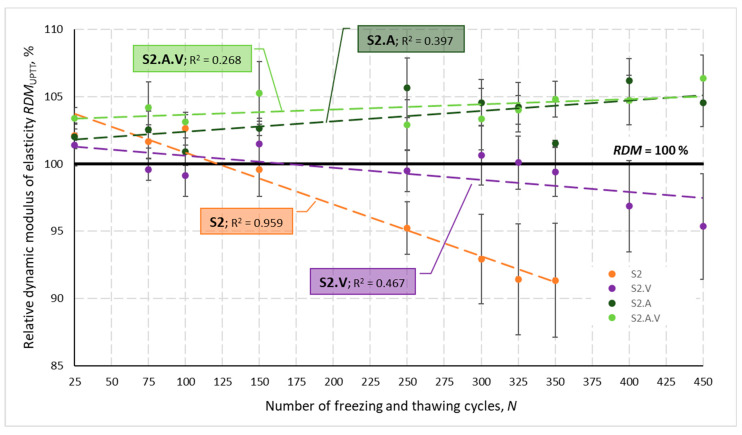
Mean relative dynamic modulus of elasticity for S2, S2.V, S2.A and S2.A.V concretes after *N* cycles of freezing and thawing.

The concrete containing AEA met all the criteria for frost-resistant concrete after 300 test cycles:

- A slight increase in strength was achieved in the case of concrete S2.A (3.3%) or a slight decrease (3.1%) for concrete S2.A.V—Table 7;
- *RDM*_UPPT_ values were > 100% regardless of the number of cycles—Figure 16;
- The weight loss of the specimens was negligible up to 0.1%.

The increased amount of air in the concrete mix, compared to the non-air-entrained concretes S2 and S2.V, resulted in the following:

- Very high loss of compressive strength (49.3 and 32.6%), which excluded the frost resistance of concrete, turned into only a few percent decrease in strength or even a slight increase in strength after the frost resistance test—Table 7;
- The trend for a continuous decrease in the value of *RDM*_UPPT_ obtained for concretes without AEA changed into the opposite tendency, i.e., a continuous, although slight, increase in *RDM*_UPPT_—Figure 16.

The Student’s *t*-test comparing the mean compressive strength of concrete and the value of *RDM*_UPTT_ after 300 cycles of freezing and thawing showed for concrete pairs S2 and S2.A, as well as S2.V and S2.A.V, that these concretes come from a population with different mean values. Thus, the effect of AEA admixture on frost resistance was significant.

As expected, the mean compressive strength of concrete after 28 days of curing was significantly lower than that of concrete without AEA:

- By 18.7 N/mm^2^ between S2 and S2.A concrete;
- By 8.0 N/mm^2^ between S2.V and S2.A.V concrete.

The loss of compressive strength of concrete with AEA after 28 days correlated with an over 3% decrease in the ultrasonic pulse velocity.

After 128 days, the compressive strength in relation to non-air-entrained concrete was (Table 6)

- Lower by 14.3 N/mm^2^ in the cases of concretes S2 and S2.A;
- Almost identical for concretes S2.V and S2.A.V.

The strength gain between 28 and 128 days of curing was significantly higher compared to concrete without AEA (Table 7):

- The relative increase in strength of S2.A air-entrained concrete was 31.1%, while for S2 concrete without AEA, it was 17.4%;
- The relative increase in strength of S2.A.V air-entrained concrete was 11.5%, while for S2.V concrete without air-entraining admixture was 4.4%.

The S2 and S2.V concretes were characterized by similar compressive strengths after 28 days of curing, which was proved by the statistical test for the equality of the mean values in the population. Similarly, in the case of S2.A and S2.A.V concretes, the Student’s *t*-test showed the equality of the mean values in the population (despite the 16% difference). It follows that the admixture of VMA has no significant influence on this strength. The statistical tool also confirmed the non-obvious influence of VMA on the strength of concrete after 128 days of curing. In the case of a pair of concretes S2 and S2.V, the mean values can be considered different, indicating the existence of the VMA influence. However, this cannot be stated in the case of S2.A and S2.A.V concretes, where the mean strengths should be considered equal.

As mentioned earlier, the authors did not find any information on the influence of the VMA admixture on concrete parameters other than rheological properties.

### 4.4. Directions of Future Works

The tests analyzed in publications [6,7,50,53] indicate that the self-compacting of concrete due to the use of superplasticizers has a positive effect on the frost resistance of concrete. The study [53] describes the internal frost resistance tests of self-compacting concrete and concrete compacted by vibration containing from 5.5 to 8% of the air in the mix. More significant decreases in the relative dynamic modulus of elasticity, weight loss of specimens and increase in the length of specimens made of vibrated concrete were demonstrated.

The tests proved that air-entrained concrete is frost-resistant due to the side effect of the superplasticiser. However, this does not always have to be the case as there are many different types of superplasticisers with very different compositions, also with anti-foam admixtures. If the type of air-entraining superplasticizer were to be replaced, the porosity structure would be inadequate for preserving the frost resistance of the concrete. It should be emphasized that the necessity to use air-entraining to ensure the frost resistance is proved by the results of studies presented, for example, in the publication [54].

Attention should be paid to the low number of tests of the mechanical properties of concretes containing admixtures. Research is mainly carried out on the influence of these admixtures on the rheological properties of concrete mixes.

There are no findings regarding the impact of additional admixtures used in concrete to clearly state whether the modification through admixtures is neutral concerning concrete’s mechanical properties and durability. The effect of cement type and the negative effect of a retarding admixture on concrete frost resistance in the presence of deicing salts was found in tests described in the article [3]. Retarding admixtures also delay the process of increasing concrete strength. Thus, the verification of concrete frost resistance carried out in a standardised period may qualify such concrete as frost-resistant. After 90 days of curing such concrete, it is probably that it would achieve frost resistance. Appropriate experimental studies should be carried out to resolve this issue. In addition, other admixtures, such as hardening and set-accelerating admixtures, shrinkage-reducing admixture and comprehensively acting in this area, may also affect the air-entraining of self-compacting concrete and the development of its strength and, therefore, frost resistance.

An important and poorly understood parameter is the influence of the admixture dosage on the properties of hardened concrete.

It is also crucial to correlate the results of concrete strength and frost resistance tests with destructive and non-destructive methods, facilitating the analysis of the influence of given admixtures during concrete maturation. This task is extremely complicated when the composition of self-compacting concrete contains pozzolanic additives that thicken the concrete structure during its maturation. Therefore, the analysis of the influence of admixtures on mechanical properties and durability seems to be an extremely complex issue and requires further research in this area.

Destructive tests aimed at assessing frost resistance are carried out in a uniaxial state of stress. Many elements of concrete structures are in a complex state of stress. Therefore, the following question arises: does the state of stress affect the internal frost resistance of concrete? To the best of the authors’ knowledge, the first testing of frost resistance in a complex stress state was published in [46]. It seems that this type of research should be extended to concretes of various compositions, containing mineral additives and/or chemical admixtures.

## 5. Conclusions

The following conclusions concern the tests of self-compacting concrete with the mean compressive strength no less than 55 N/mm^2^, carried out in the range described in the article.

The influence of admixtures and their combinations on internal frost resistance after 300 cycles of freezing and thawing and the compressive strength of concrete matured at room temperature was analysed.

The second group of conclusions concerns the compatibility of measures and criteria for estimating the frost resistance of concrete with the methods used in these tests. Their confirmation may be the results of tests [55] obtained after 300 freezing and thawing cycles.

### 5.1. The Influence of Chemical Admixtures on Frost Resistance and Strength of Concrete

- The superplasticizer based on polycarboxylic ethers unintentionally caused air-entraining to the concrete mix to about 8%. This resulted in the frost resistance of concrete after 300 freeze–thaw cycles. A potentially dangerous side effect of air entrainment was about 17% lower compressive strength compared to concrete with the same base composition, which contained a superplasticiser and did not demonstrate the above-mentioned negative effect.
- The addition of AFA to the unintentionally air-entrained concrete caused a decrease in the amount of air by about 5% in the concrete mix and about 2.5% of air voids in hardened concrete. The concrete with AFA still met all the frost resistance criteria used in tests. The decrease in air content did not result in the expected increase in the compressive strength. This effect may be due to the negative effect of the AFA admixture components (fatty alcohol ester) on cement hydration.
- The air-entrained concrete had the expected positive and negative effects. The air content increased to 5% in concrete mix and to near 4% in hardened concrete. According to the criterion of the change in compressive strength, the air entrainment of concrete mixtures caused the frost resistance of concrete, which was not frost-resistant. The negative effect was the 8–20% reduction in the compressive strength.
- The effect of VMA admixture was not evident. The influence of VMA in unintentionally and intentionally air-entrained concrete on internal frost resistance and compressive strength determined after 28 days of curing was insignificant. The influence on the compressive strength after 128 days of curing in non-air-entrained concrete was statistically significant.

### 5.2. Commensurability of the Criteria for Assessing the Internal Frost Resistance of Concrete

- In the case of non-air-entrained concretes, the obtained frost resistance indexes indicated both a frost resistance and lack of frost resistance, depending on the assessment method and the corresponding criterion. The mentioned concretes did not meet the criterion of permissible loss of compressive strength; the decrease in strength after 300 cycles of the frost resistance test reached 50%.
- The indicator in the form of the relative dynamic modulus of elasticity correctly described the damage caused by the impact of low temperatures only qualitatively. In the case of non-air-entrained concretes, a constant decrease in the value of *RDM*_UPTT_ was obtained, but the decrease was disproportionately low concerning the loss of concrete strength.
- There was no loss in weight of the non-air-entrained concrete specimens, despite a large reduction in strength. The criterion of sample mass change should only determine the resistance to surface damage caused by frost.
- Internal frost resistance, understood as resistance to degradation of the internal structure of concrete, as shown in this research, is best assessed using destructive methods. The decisive criteria for internal frost resistance should be the change in concrete strength and/or the static modulus of elasticity, which directly reflect the mechanical damage of concrete.

## Figures and Tables

**Table 1:** Concrete composition.

Cement CEM II B-S 32.5 R (C), kg/m^3^	Limestone Powder (LP), kg/m^3^	w/c	w/b w/(C+LP)	Sand 0–2 mm, kg/m^3^	Gravel 2–8 mm, kg/m^3^	Volume of Cement Paste, %
442.4	190.0	0.45	0.31	693.2	866.5	41.0

**Table 2:** Tested concretes, types and effects of admixtures used.

Concrete Symbol	Admixtures ^(a)^	The Effect of Admixtures
S1	SP.1 „air-entraining”	Increasing the degree of fluidity and unintentional air-entraining
S1.F	SP.1 + AFA	Increasing the degree of fluidity and elimination of unintentional air-entraining due to the action of SP.1
S1.F.V	SP.1 + AFA + VMA	Increasing the degree of fluidity, elimination of unintentional air-entraining and preventing segregation due to the action of SP.1 and AFA
S2	SP.2	Increasing the degree of fluidity
S2.V	SP.2 + VMA	Increasing the degree of fluidity and preventing segregation due to the action of SP.2
S2.A	SP.2 + AEA	Increasing the degree of fluidity and intentional air-entraining
S2.A.V	SP.2 + AEA + VMA	Increasing the degree of fluidity, intentional air-entraining and preventing segregation due to the action of SP.2 and AEA

^(a)^ SP—superplasticiser; AFA—anti-foam admixture; VMA—viscosity-modifying admixture; AEA—air-entraining admixture.

**Table 3:** The dosage of admixtures by weight of the total binder, % [25].

Concrete Symbol	Type of Admixture				
	SP1	SP2	AFA	VMA	AEA
S1	0.67	-	-	-	-
S1.F	0.67	-	1.80	-	-
S1.F.V	0.67	-	1.80	0.25	-
S2	-	1.33	-	-	-
S2.V	-	1.53	-	0.25	-
S2.A	-	1.16	-	-	0.04
S2.A.V	-	1.51	-	0.25	0.08

**Table 4:** Consistency and air content in concrete mixes modified with admixtures [26,27].

Concrete Symbol	Flow Spread *D*, mm	Flow Time *T*_500_, s	Air Content *A*_c_, %
S1	730	3	8.0
S1.F	705	2	2.7
S1.F.V	710	5	3.1
S2	715	2	2.1
S2.V	710	4	2.5
S2.A	640	3	5.0
S2.A.V	690	4	5.0 ^(a)^

^(a)^ the same amount of air in the concrete mix, despite the double amount of AEA, results from a larger amount of superplasticiser (Table 3), an increased degree of fluidity, and therefore, a stronger tendency to escape air bubbles from the mix.

**Table 5:** Relative weight loss after 300 cycles of freezing and thawing, Δ*m*_rel_.

Concrete Symbol	Δ*m*_rel_, % ^(a)^
S1	0.00 (-)
S1.F	0.89 (7.25)
S1.F.V	0.52 (10.5)
S2	0.00 (-)
S2.V	0.00 (-)
S2.A	0.03 (6.25)
S2.A.V	0.10 (10.0)

^(a)^ in brackets coefficient of variation value is given in percent.

**Table 6:** Mean compressive strength of non-frozen concrete after 28 and 128 days of curing and concrete after 300 cycles (100 days) of freezing and thawing.

Concrete Symbol	Mean Compressive Strength of Concrete ^(a)^		
	not Frozen after		after 300 Cycles of Freezing and Thawing *f*_c.mv.300FT_, N/mm^2^
	28 days *f*_c.mv.28d_, N/mm^2^	128 days *f*_c.mv.128d_, N/mm^2^	
S1	61.7 (3.22)	69.4 (4.86)	71.5 (5.46)
S1.F	61.9 (5.57)	69.7 (6.19)	70.0 (6.48)
S1.F.V	57.5 (7.81)	66.5 (9.94)	64.8 (8.61)
S2	74.6 (2.14)	87.6 (4.40)	44.4 (9.48)
S2.V	73.1 (4.24)	76.3 (6.73)	51.4 (4.87)
S2.A	55.9 (6.93)	73.3 (8.16)	75.7 (7.01)
S2.A.V	65.1 (6.04)	72.6 (6.92)	70.3 (2.70)

^(a)^ in brackets coefficient of variation value is given in percent.

**Table 7:** Absolute and relative changes in compressive strength of tested types of concrete.

Concrete Symbol	Increase in Strength of Unfrozen Concrete between 28 and 128 Day		Decrease in Strength after 300 Cycles of Freezing and Thawing ^(a)^	
	Absolute Δ*f*_c.abs.d_, N/mm^2^	Relative Δ*f*_c.rel.d_, %	Absolute Δ*f*_c.abs.300FT_, N/mm^2^	Relative Δ*f*_c.rel.300FT_, %
S1	7.7	12.5	−2.1	−3.0
S1.F	7.8	12.6	−0.3	−0.4
S1.F.V	9.0	15.7	1.7	2.6
S2	13.0	17.4	43.2	49.3
S2.V	3.2	4.4	24.9	32.6
S2.A	17.4	31.1	−2.4	−3.3
S2.A.V	7.5	11.5	2.3	3.1

^(a)^ negative values indicate an increase in compressive strength.

## Data Availability

The data presented in this study are available on request from the corresponding author.
